# The great potential of entomopathogenic bacteria *Xenorhabdus* and *Photorhabdus* for mosquito control: a review

**DOI:** 10.1186/s13071-020-04236-6

**Published:** 2020-07-29

**Authors:** Wellington Junior da Silva, Harry Luiz Pilz-Júnior, Ralf Heermann, Onilda Santos da Silva

**Affiliations:** 1 Department of Microbiology, Immunology and Parasitology, Institute of Basic Health Sciences, Universidade Federal do Rio Grande do Sul, Rua Sarmento Leite 500, Porto Alegre, RS 90050-170 Brazil; 2 Institut für Molekulare Physiologie, Mikrobiologie und Weinforschung, Johannes-Gutenberg-Universität Mainz, Johann-Joachim-Becher-Weg 13, 55128 Mainz, Germany

**Keywords:** Entomopathogenic bacteria, *Aedes aegypti*, Mosquito-borne arboviruses, *Xenorhabdus nematophila*, *Photorhabdus luminescens*, Biological control

## Abstract

The control of insects of medical importance, such as *Aedes aegypti* and *Aedes albopictus* are still the only effective way to prevent the transmission of diseases, such as dengue, chikungunya and Zika. Their control is performed mainly using chemical products; however, they often have low specificity to non-target organisms, including humans. Also, studies have reported resistance to the most commonly used insecticides, such as the organophosphate and pyrethroids. Biological control is an ecological and sustainable method since it has a slow rate of insect resistance development. Bacterial species of the genera *Xenorhabdus* and *Photorhabdus* have been the target of several research groups worldwide, aiming at their use in agricultural, pharmaceutical and industrial products. This review highlights articles referring to the use of *Xenorhabdus* and *Photorhabdus* for insects and especially for mosquito control proposing future ways for their biotechnological applicability. Approximately 24 species of *Xenorhabdus* and five species of *Photorhabdus* have been described to have insecticidal properties. These studies have shown genes that are capable of encoding low molecular weight proteins, secondary toxin complexes and metabolites with insecticide activities, as well as antibiotic, fungicidal and antiparasitic molecules. In addition, several species of *Xenorhabdus* and *Photorhabdus* showed insecticidal properties against mosquitoes. Therefore, these biological agents can be used in new control methods, and must be, urgently considered in short term, in studies and applications, especially in mosquito control.
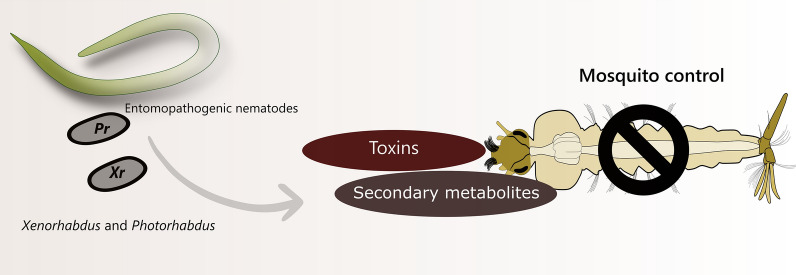

## Background

It is widely known that various species of mosquitoes can transmit pathogens that cause debilitating injuries in world populations, often endangering the lives of millions of people. Among the mosquito-borne viruses are dengue, chikungunya [1], West Nile virus, yellow fever [2] and Zika [3].

The presence of chikungunya virus has been notified in more than 45 countries, highlighting the epidemic that occurred in India in 2005 with 1015 confirmed cases [4]. In Brazil, the first autochthonous cases were reported in September 2014, with subsequent emergence of cases in several regions, in a short period of time [5]. In 2016, 63,810 cases of chikungunya were confirmed in this country [6].

West Nile fever virus was first isolated in 1937 from an infected person in Uganda, Africa [7]. After that, sporadic transmission was observed in more temperate parts of Europe and endemic in tropical areas of Africa, northern Australia and South Asia. The virus was introduced in North America in 1999, which spread and became a public health problem [8]. After its introduction in America, in 2010 approximately 1.8 million people have been infected, resulting in 1308 deaths [9].

Since 2016 there has been the re-emergence of the Zika virus, with outbreaks of transmission by mosquitoes, causing a threat to public health worldwide [3, 10–14]. Regarding dengue virus, it is expanding globally, being present in more than 100 countries [15], with about 2.5 billion people living in an infection risk area [16–18]. Currently this virus is found on all continents [13, 18]. Because of this global viral spread, dengue has become an important public health problem [15, 19], being a threat to approximately 390 million people [3, 16].

According to Laughlin et al. [19], considering complications of dengue such as hemorrhagic fever and shock syndrome, is among the most important re-emerging infectious diseases in the world. This arbovirus is the one with the greatest impact on human morbidity and mortality compared to other arboviruses [20] due to the high virulence of the etiological agent [21]. Their four viral serotypes can be transmitted by females of *Aedes aegypti* mosquitoes (Diptera: Culicidae), and, to a lesser extent, *Aedes albopictus* [3, 12, 14, 22].

*Aedes aegypti* has a daylight hematophagous behavior and is extremely anthropophilic and endophilic, often being found in urban and suburban environments, where the life-cycle occurs with great proximity to humans [23]. Female oviposition occurs preferably in clean water present in artificial containers [24]. *Aedes albopictus*, popularly known as the Asian tiger, is also a daylight hematophagous mosquito, and can be found competing with *Ae. aegypti* in natural and artificial containers outside of houses. Also, it has expanded its habitat to temperate areas in urbanized regions due to intense climate change [18, 25].

These often-devastating arboviruses seem to continue to affect millions of people worldwide [26]. Therefore, vector control, whether in immature or adult stages, is a crucial strategy for preventing the expansion of these diseases [26, 27].

For the production of this narrative review, keywords were chosen: *Xenorhabdus*; *Photorhabdus*; insect and mosquito control; arbovirus and *Aedes*. All articles that contained information relevant to the purpose of the review were selected and used for their construction. As we propose that these bacterial species can be used to control mosquitoes, no temporal delimitation was made for the inclusion of the articles, aiming at a greater range of results that could be used.

## Current vector control and their resistance to some compounds

Vector control is a method of extreme relevance to minimize the transmission of disease agents by mosquitoes [28, 29]. Therefore, limiting the impact of mosquito-borne diseases is an important goal for global public health agencies [26]. For the control of culicids several methods have been used, which result in reduction of population density, reduction of life span, or impediment of contact with the use of repellent compounds [30].

The methods for genetic control are in the study phase for *Ae. albopictus* and *Ae. aegypti*, also requiring considerations on the possibility of its implementation [30–32]. Thus, chemical control is generally considered the first method of choice [27].

In addition, several strategies for combating *Aedes* spp. have been used, such as the elimination of potential breeding sites, biological and chemical control with the use of repellents (contact precaution) and application of synthetic insecticides [14, 29, 30].

Among the most used compounds, organophosphates (temephos and fenthion) and growth inhibitors (diflubenzuron and methoprene) for larval control [14, 33–35]. However, due to the high frequency of use of these compounds, several populations of *Aedes* spp. have become resistant over the years [14, 26, 29, 36–38].

Chemical control may have disadvantages, such as effects on non-target organisms, environmental pollution, in addition to the development of insecticide resistance [27, 39–43]. Furthermore, repeated doses and high doses of chemical insecticides can cause an imbalance between the culicid population and their natural enemies, and also cause toxic effects on the environment and small mammals that co-inhabit the surrounding area [40]. Thus, it is necessary to reduce the use of chemicals and develop ecological products for the control of vector mosquitoes [44].

## Biological control of vectors

The nutrition source of *Aedes* larvae comes from decaying organic matter, rich in bacteria, fungi and protozoa present in natural or unusual containers [45]. Some bacterial species may produce secondary toxins and metabolites capable of inducing larval death. Thus, symbiotic bacteria possibly cause pathogenicity after being ingested by mosquitoes [14].

Biological vector control is an ecological and sustainable method since it has a slow rate of insect resistance development [27]. Insecticide activities have been investigated in several microorganisms, including bacteria [46, 47], protozoa [48] and fungi [49]. The Gram-positive bacterium *Bacillus thuringiensis israelensis* (*Bti*) has been widely used as a biolarvicide in aquatic environments for mosquito control [50] and some species of the family Simuliidae [51–53]. In addition, *Bti* was an excellent candidate for fly control due to its entomopathogenic activities [50, 54], being widely used in recent years as researchers developing studies to improve its effectiveness [44].

The World Health Organization recommends the use of biolarvicides derived from *Bti* and *Bacillus sphaericus* (syn. *Lysinibacillus sphaericus*) to control mosquito larvae, because they are alternative products that do not cause harm to the environment. Nevertheless, there are few options for bacterial larvicides available [55, 56]. The use of *Bti* also presented an impact on the prevalence of malaria [57]. Due to its mechanism of action, with release of toxins in the midgut of the larvae, the development of insect resistance can be hampered [26]. Although some authors have already recorded the occurrence of mosquitoes resistant to *Bti* [58, 59].

Other biological agents have been described for the control of *Aedes* species, such as: (i) fungi *Metarhizium anisopliae* and *Beauveria bassiana* [49, 60, 61]; (ii) protozoan *Acanthamoeba polyphaga* [48]; (iii) the copepod *Macrocyclops albidus* [62]; (iv) as well as bacteria of the genera *Xenorhabdus* and *Photorhabdus* [13, 29, 44, 50, 63–65].

## Symbiotic nematoid bacteria and insect control

The study of bacterial species of the genera *Xenorhabdus* and *Photorhabdus* has been the target of several research groups, aiming at their use in agricultural, pharmaceutical and industrial products [43, 66–68]. The interest in studying these bacteria is justified by some evidence available in the literature, such as: (i) having genes that are capable of encoding low molecular weight secondary toxins and metabolites with insecticide activities [43, 69–71], antibiotic [43, 69, 72–74], antifungals [43, 69] and antiparasitic [69, 75–78]; (ii) laboratory research points to the success of these bacteria in pest control [27, 79]; (iii) *Photorhabdus luminescens* releases toxins with activities in the insect intestinal epithelium [59, 80]; (iv) *P. luminescens* in conjunction with *B. thuringiensis kurstaki* inhibits the growth of *Spodoptera littoralis* [81]; (v) *Xenorhabdus ehlersii* protein (XeGroEL) is effective against *Galleria mellonella* [82, 83]; (vi) acaricide and antibacterial activities have been reported for *Xenorhabdus stockiae* PB09 [84, 85]; (vii) *Xenorhabdus stockiae* PB09 showed miticidal activity against *Luciaphorus perniciosus* [86]; and (viii) the supernatants of the culture of *Xenorhabdus nematophila* and *P. luminescens* prevented the feeding of ants, crickets and wasps [87, 88], among others.

Bacteria of the genera *Xenorhabdus* and *Photorhabdus* are Gram-negative, optional anaerobic, belonging to the family *Enterobacteriaceae* [89–91], which stand out for their entomopathogenic potential [29, 92]. Approximately 24 species of *Xenorhabdus* and five species of *Photorhabdus* have been described worldwide to have insecticidal properties [14, 75, 93–96].

In nature, some species such as *X. nematophila* and *P. luminescens* developed a symbiotic relationship with helminths of the class Nematoda, Steinernematidae for *Xenorhabdus* and Heterorhabditidae for *Photorhabdus* [13, 14, 27, 44, 59, 97–101]. In the nematode host, the bacteria reside in the receptacle located in the intestine [102, 103].

**Fig. 1:**
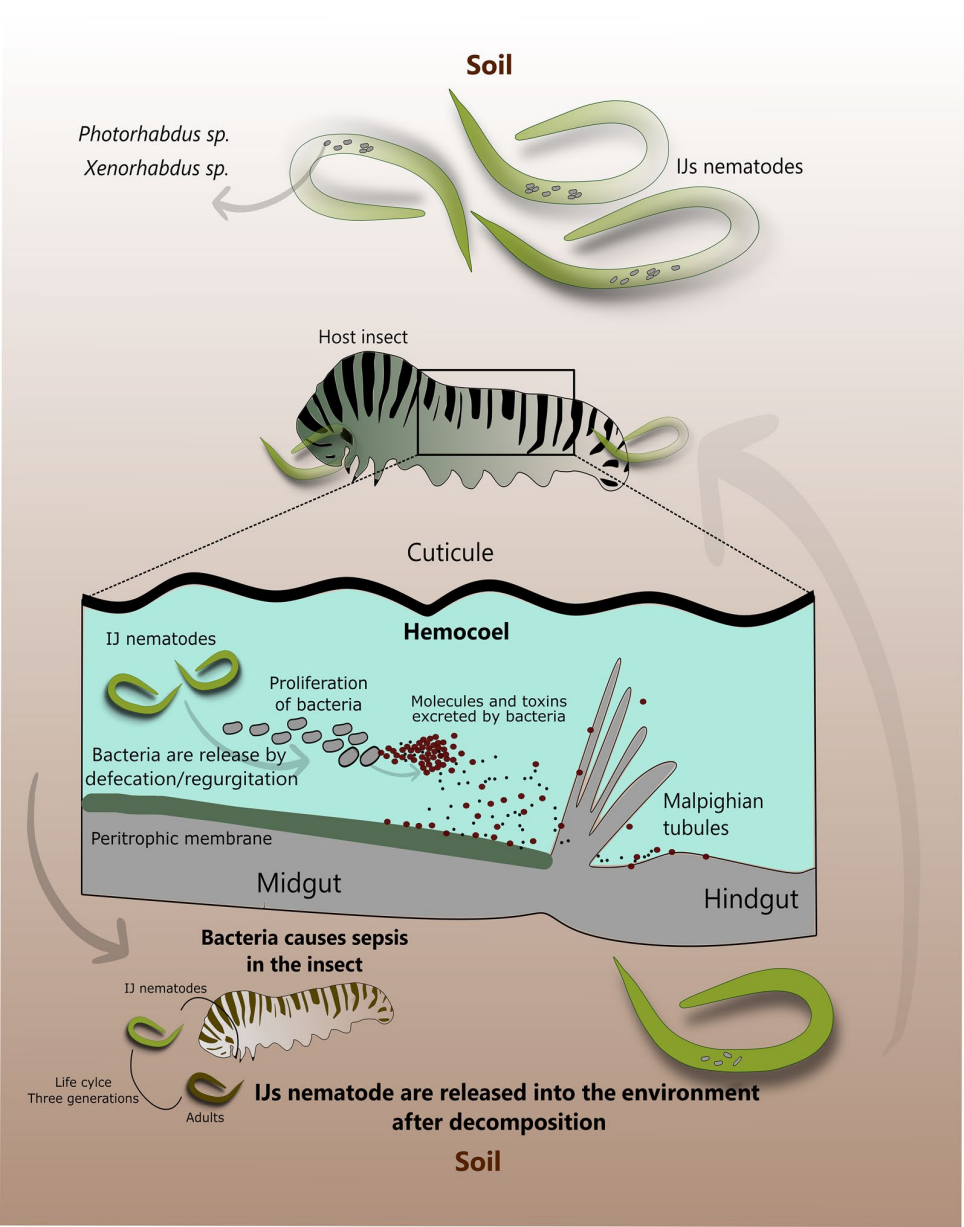
Schematic drawing of the entomopathogenic nematode cycle, with the *Photorhabdus* and *Xenorhabdus* bacteria, demonstrating their symbiosis. The nematodes roam freely in the soil until they find a host insect, in the scheme represented by a caterpillar. The nematodes, when entering the host and settling in the hemocoel, release the bacteria through defecation or regurgitation. The bacteria proliferate in the hemocoel and become infectious, when they release toxic molecules to the host, leading to their death. Nematodes use the host’s carcass to reproduce and return to the habitat carrying the bacteria, restarting the cycle until the nematodes find a new host insect

Although these two bacterial species (*X. nematophila* and *P. luminescens*) have different evolutionary origins, the life-cycle is similar [104] and both are highly pathogenic for various insect species [13]. The cycle occurs as follows: the larvae of entomopathogenic nematodes (EPNs) live in the soil of several ecological systems searching for insect larvae as prey [105]. When found, it penetrates the insect’s body through natural openings, such as the mouth, anus or spiracles [69], or they directly reach the hemocoel by boring a hole into the insect’s skin, where symbiotic bacteria will be released by regurgitation (e.g. Heterorhabditidae) and defecation (e.g. Steinernematidae) [106, 107]. Once inside the hemocoel, the bacteria actively replicate, and release compounds that have the potential to suppress the immune response of the host insect, this being a protection strategy for symbiosis with the nematode [108, 109]. Taking into account the immunosuppressed state of the host, bacteria multiply in the hemocoel, initiating a fatal septicemia for the insect [99], causing its death in about 24 to 48 hours. Soon after, the carcass is bio-converted by bacteria, forming a rich food source for the nematodes as well as for themselves. Nematoid larvae grow and reproduce, giving rise to new youth stages. Furthermore, reproduction and development of the nematodes is actively supported by the bacteria by a yet unknown mechanism [14, 29, 44]. With food depletion, symbiotic association occurs again and the new helminth larvae adopt a free life phase (soil), where they actively transport their endosymbiotic bacteria and searching for new insect hosts [13, 99, 101, 110] (Fig. 1).

The symbiotic relationship, for example, between *Steinernema* and *Xenorhabdus* infecting insects, is mutually beneficial for the helminth-bacterium dyad, because carcasses become a nutritional source and breeding site for both the helminths and the bacteria. Furthermore, it is important to highlight that endosymbiotic bacteria are essential for the death of the next insect that will be parasitized, playing a crucial role in the survival of these nematodes [111].

In terms of specificity, *X. nematophila*, *X. hominickii* and *Photorhabdus temperata temperata* were isolated from *Steinernema carpocapsae*, *Steinernema monticolum* and *Heterorhabditis megidis*, respectively [103, 112, 113]. On the other hand, *X. nematophila* is not able to colonize *Steinernema scapterisci* [114]. Yooyanget et al. [14] described the importance of studying the species-specific identification in the mutualism between nematodes and entomopathogenic bacteria to obtain information about their diversity, as well as distribution in space, to additional studies of bioactive compounds that can be used in mosquito control.

Other differences in nematode-bacterial interactions have been described. For example, *Xenorhabdus innexi* usually associates with *S. carpocapsae*, but with only one to five cells in the intestine of the nematode. *Xenorhabdus nematophila* colonizes the entire intestinal receptacle [115]. *Photorhabdus asymbiotica* is a species pathogenic to humans. However, *Photorhabdus asymbiotica australis* maintains an entomopathogenic symbiosis with nematodes of the genus *Heterorhabditis* [59, 100, 116].

Regarding the interaction of the bacteria with insects, in the moth *Manduca sexta*, infected with *Photorhabdus* and *Xenorhabdus*, colonization occurs primarily in the anterior portion of the midgut and then spreads to the posterior intestine [29]. *Photorhabdus* sp. secrete toxins that are able to destroy the intestinal epithelium of this insect, resulting in the interruption of the host’s feeding process [29]. It is also noteworthy that in conjunction with insect feeding the virulence of the *Xenorhabdus* species is altered [29, 117].

*Xenorhabdus* are pathogenic to insects even in the absence of nematodes, as they are able to kill them after experimental injection. Thus, several studies are being developed in order to use *Xenorhabdus* for pest control [118]. Plants expressing certain genes of *X. nematophila* can become resistant to some insect species [119, 120]. For example, oral ingestion of transgenic *Arabidopsis thaliana* (expressing a gene from the *P. luminescens* toxin complex) was highly toxic to *M. sexta*, conferring plant resistance to insects and their oral mortality [121].

When released into the hemolymph of various insects, *Photorhabdus* bacteria are highly pathogenic [122]. It is important to emphasize that so far, no resistance to these bacteria has been reported in insect populations [58, 59, 80]. Some toxins of *P. luminescens* have a mode of action that differ from the toxins of *B. thuringiensis* and, these toxins for insect control can serve as potential alternatives [110, 123].

Even at low doses, *X. nematophila* demonstrates high toxicity to larvae of *Galleria mellonella*. After inoculation of bacteria (independent dose), the colony reached more than one million colony-forming units (CFU’s) in a short period of time (≤ 24 hours). A trial conducted with adults of *Drosophila melanogaster* inoculated with *X. nematophila* showed similar results, with rapid death of the insects, but the colony reached one million CFU’s in a shorter time (≤ 18 hours). The same adults of *D. melanogaster* seemed to be highly resistant to *X. innexi* [114], which is also not effective in the death of larvae of *M. sexta*, while *X. nematophila* is highly toxic to both insects [43].

The insecticide activity of *Xenorhabdus* and *Photorhabdus* species is related to protein production [66, 110, 124, 125] and secondary metabolites [13, 69, 75, 126–128]. The secretion of toxins of high molecular weight by *P. luminescens* and *X. nematophila* plays an important role in insect mortality [29, 66, 125]. As described, pathogenicity is related to cell replication and production of toxins in the hemocoel causing histological injury, and septicemia [117].

**Fig. 2:**
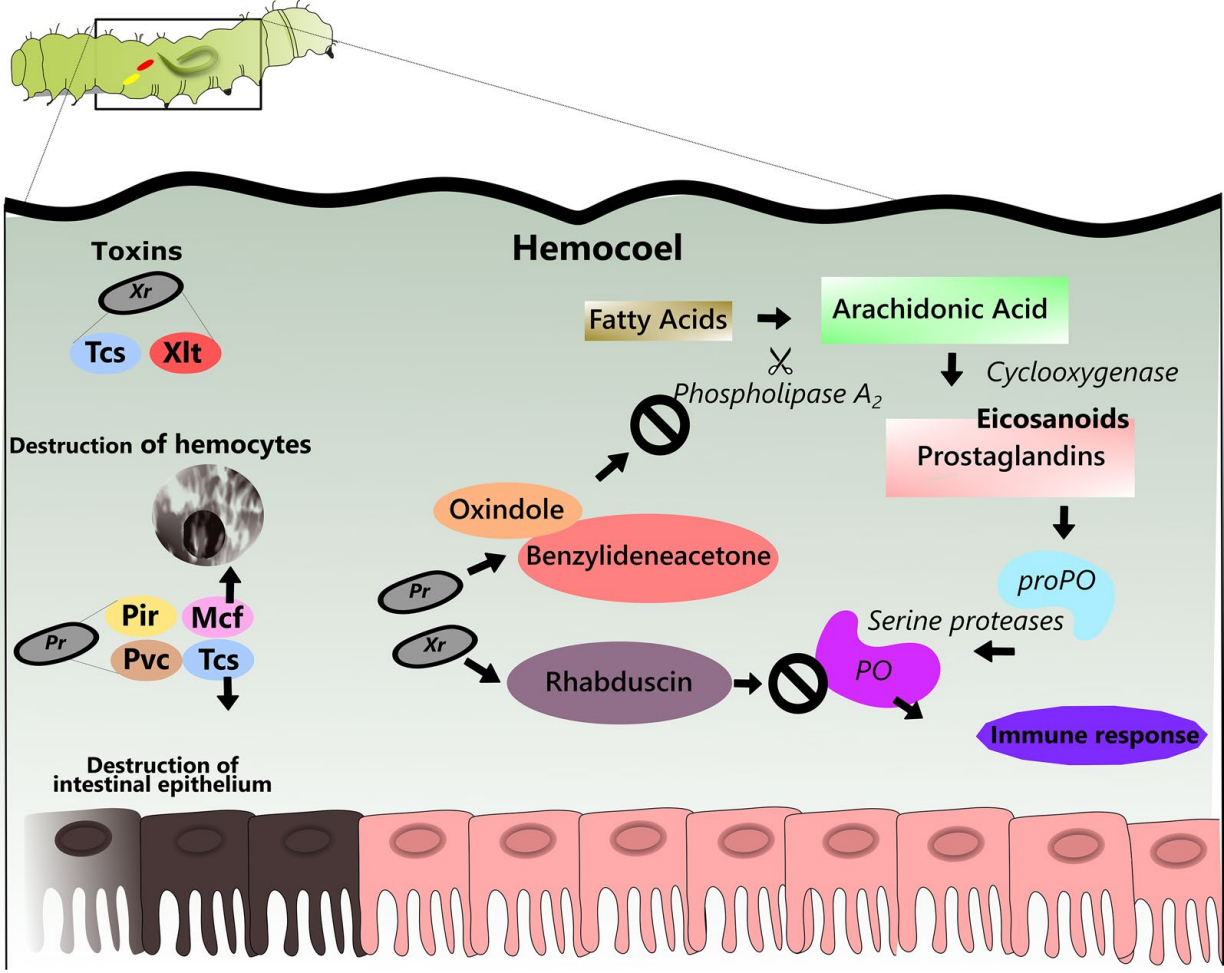
Schematic drawing of toxins and mode of action of some compounds produced by the bacteria *Xenorhabdus* and *Photorhabdus*. *Xenorhabdus* can produce toxin complexes that induce immunosuppression in insects by inhibiting eicosanoid synthesis. The *Xenorhabdus* lipoprotein toxin produced by *X. innexi* has toxic properties against culicids. *Photorhabdus* also produces toxin complexes, which have activity directly in the intestinal epithelium of insects, leading to their destruction. Make caterpillars floppy causes apoptosis in hemocytes in the hemocoel. *Photorhabdus* virulence cassettes, encode genes that are toxic action against some species of lepidopterous. Insect-related protein is highly toxic and is similar to δ endotoxins of *Bacillus thuringiensis*. *Photorhabdus* can produce toxins that directly affect Phospholipase A_2_, while *Xenorhabdus* produces toxins that inhibit phenoloxidase produced through prophenoloxidase, directly affecting the insect’s immune system. *Abbreviations*: Xr, *Xenorhabdus*; Tcs, toxin complexes; Xlt, *Xenorhabdus* lipoprotein toxin; Pr, *Photorhabdus*; Mcf, make caterpillars floppy; Pvc, *Photorhabdus* virulence cassettes; Pir, insect-related protein; PO, phenoloxidase; proPO, prophenoloxidase

Samples of *Xenorhabdus* produced toxins (Tcs) that induce immunosuppression in insects by inhibiting eicosanoid synthesis [108, 129]. *Xenorhabdus nematophila* produces about eight suppressor metabolites of insect immunity [109]. *Xenorhabdus budapestensis* produces hybrid compounds called fabclavins, which exhibit antibiotic and insecticide activities [69, 73, 130, 131]. Some species of *Photorhabdus* produce a variety of toxins including Tcs (toxin complexes), Mcf (make caterpillars floppy), Pvc (*Photorhabdus* virulence cassettes) and Pir (insect-related protein) [132]. The Tcs destroy epithelial cells from the middle intestine of insects, similar to δ-endotoxin of *B. thuringiensis* and acting on the actin cytoskeleton by the ADP-ribosyltransferases TccC3 and TccC5 in *P. luminescens* [132, 133]. On the other hand, Mcf promotes hemocytes apoptosis in the hemocoel [134]. It was also observed that *M. sexta* and *G. mellonella* are susceptible to Pvc [135] (Fig. 2).

Predictive genes of toxins, proteases and haemolysins are abundant in the TT01 strain of *P. luminescens laumondi*, which may be involved in pathogenicity [136]. In this strain, Pir proteins are related to insect death. The proteins are encoded by genes *plu4093*-*plu4092* for PirA and *plu4437*-*plu4436* for PirB, respectively. The corresponding proteins have similarity with δ endotoxins of *B. thuringiensis* and with a growth regulatory protein of *Leptinotarsa decemlineata. Photorhabdus luminescens* and *P. asymbiotica* Pir proteins heterologously produced in *Escherichia coli* have the ability to cause the death of larvae of *G. mellonella*, with high toxicity [137].

Previously, Bode [69] suggested further studies on bacterial secondary metabolites due to the possibility of their use as new control agents of agricultural pests and/or vector insects, such as mosquitoes. Some secondary metabolites produced by *X. nematophila* and *P. temperata temperata* are supposed to be responsible for the suppression of the enzyme phospholipase A_2_, causing impairment in the eicosanoid biosynthesis [138] and consequently in the immune response of insects [139]. Two bacterial metabolites that can inhibit phospholipase A_2_ are oxindole and benzylideneacetone in insects [138, 140]. Such metabolites have also been reported as potentiators of toxicity of *B. thuringiensis* against lepidopterans and coleopterans, acting in suppressing the immune response of these insects. [109, 141]. In cultures of *X. nematophila* and *P. temperata temperata*, seven metabolites with the function of inhibiting phospholipase A_2_ were identified [138].

The phospholipase A_2_ enzyme has a function of catalyzing fatty acids (mainly acynic acid) that will later be oxygenated by cyclooxygenase and lipooxygenase enzymes for the production of prostaglandins and leukotrienes, respectively, which are mediators of the immune response in insects [44, 138, 139, 142, 143]. Prostaglandins induce the release of profenoloxidase (proPO) of oenocytoids in plasma, for the formation of active phenoloxidase (PO) [144] which, in insects, is indispensable for the execution of humoral and cellular immune responses [44, 145].

Another common mechanism of *Xenorhabdus* in insect immunosuppression is direct suppression of the PO enzyme that is present in hemolymph in the inactive proPO form. PO is activated by proPO cleavage by protease serines [146]. Secretion of rebduscin by *X. nematophila* inhibits the activation of PO [138, 147] (Fig. 2).

*Xenorhabdus innexi* in association with *Steinernema scapterisci* is effective in killing some insects [114, 148, 149], mainly crickets [43]. In order to verify the immunosuppression capacity of insects by this bacterial species, Kim et al. [43] evaluated the inhibition capacity of PO activation in *M. sexta*. However, there was no secretion of immunosuppressive metabolites that could be detected in the trial performed with cell cultures. The same authors identified that the genome of *X. innexi* has a reduction in gene complements predicted to encode virulence determinants compared to other species of the same genus. However, *X. innexi* secret Xlt (*Xenorhabdus* lipoprotein toxin), which is a lipopeptide with toxic properties for culicids [118, 150].

## The impact of *Photorhabdus* and *Xenorhabdus* on mosquito control

**Fig. 3:**
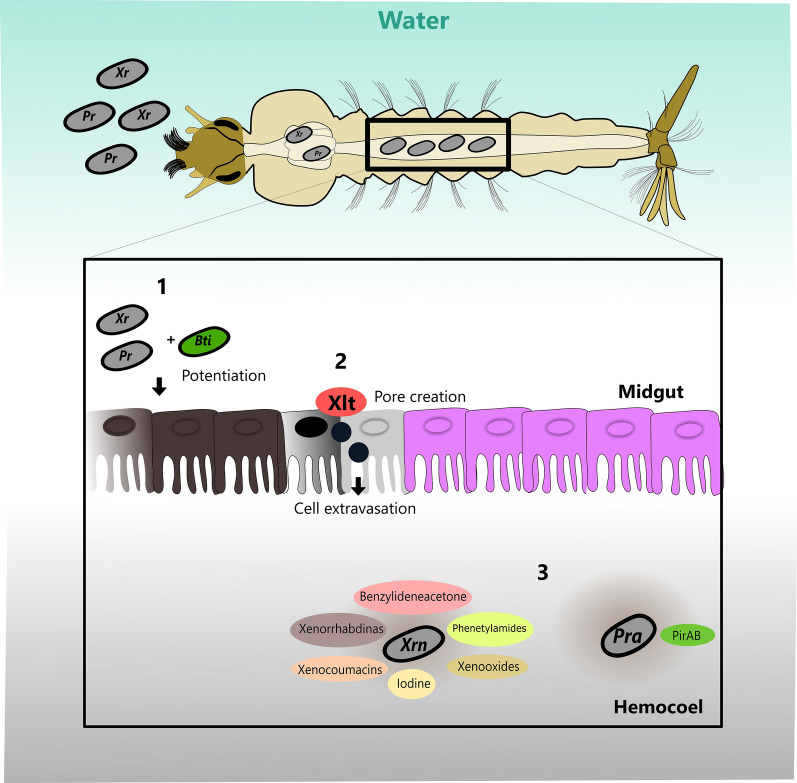
Schematic drawing summarizing the mechanisms related to *Xenorhabdus* and *Photorhabdus* for the control of culicids. **1***Xenorhabdus* and *Photorhabdus*in increase the toxic effect of Cry4Ba derived from *Bacillus thuringiensis* var. *israliensis* against *Aedes aegypti.***2***Xenorhabdus* liprotein toxin has the ability to create pores on the apical surface of cells in the anterior midgut of mosquitoes but in the anterior portion of the middle intestine, causing cell death. **3***Xenorhabdus nematophila* (*Xrn*) secretes proteins and secondary metabolites that are effective in the control of culicids, while *Photorhabdus asymbiotica* (*Pra*) produce PirAB proteins, which have already been tested on *Aedes albopictus*, *Aedes aegypti* and are toxic even by oral administration. *Abbreviations*: Xr, *Xenorhabdus*; Pr, *Photorhabdus*; *Bti*, *Bacillus thuringiensis* var. *israliensis*; Xlt, *Xenorhabdus* lipoprotein toxin; *Xrn*, *Xenorhabdus nematophila*; *Pra*, *Photorhabdus asymbiotica*

Previously, several authors described that *X. nematophila* secretes proteins and secondary metabolites that are effective in the control of culicids [69, 104], such as benzylideneacetone, [151] iodine, [72] phenethylamides and indol derivatives, [126, 128] xenorhabdins and xenooxides [128], and xenocoumacins [127] (Fig. 3).

Consequently, Gill et al. [152] described the benefits of using biological control agents with different mechanisms of action, because the synergistic effect could increase larvicidal potential and decrease the selection of resistant populations.

Ahantarig et al. [59] evaluated the larvicidal potential of *P. asymbiotica* against *Ae. aegypti*. The PirAB protein of this bacterial species were heterologously produced in *E. coli* for oral administration in larvae of the first stage of *Ae. aegypti* and *Ae. albopictus*. Mortality rates of up to 100% for the two mosquito species were observed. The concentration of 0.33 × 10^6^ cells/ml of PirAB produced by *E. coli* was sufficient for killing all *Ae. aegypti* larvae within 24 hours. The bioassay was also performed with the copepod *Mesocyclops thermocyclopoides*, a species used for larvae control (L1) of *Aedes*, whose result was negative for toxicity, as no mortality was observed.

The set of proteins that form PirAB have a greater toxic effect for *Ae. aegypti* larvae when compared with other Pir proteins in the oral bioassay [59]. It is noteworthy that the path of exposure of the larvae can interfere with the results. For example, Waterfield et al. [137] observed greater insecticide activity when injecting PirA + PirB proteins into the hemocoel of *G. mellonella*.

Shrestha et al. [153] evaluated culture fluids of five different isolates of *Photorhabdus* sp. on pathogenicity. Three days after oral ingestion of bacteria, the mortality of *Culex pipiens pallens* larvae was greater than 90%. However, of the insects tested, *P. luminescens laumondii* (TT01) could not cause mortality.

Subsequently, Silva et al. [29] evaluated the toxicity of *P. luminescens* and *X. nematophila* against *Ae. aegypti* larvae when they were feeding with these bacteria. After ingestion of bacteria, both species were toxic to the mosquito larvae within 96 hours. The authors also observed cannibalism among the larvae in all bioassays, after exposure to both bacterial species. This factor had previously been discussed by Koenraadt & Takken [154], who described several biotic and abiotic factors that can cause larval stress in aquatic environment. In this case, the presence of bacteria is discussed as a biotic factor that triggers cannibal behavior. Thus, Silva et al. [29] demonstrated that even in the absence of nematodes, the bacteria have a larvicidal effect in *Ae. aegypti* after being ingested. However, the molecular mechanism how the bacteria kill the larvae is still unknown.

In the context of mosquito control, several analyses were made aiming the establishment of new effective agents such as: (i) mixture of the culture broth of *P. temperata temperata* with *B. thuringiensis tenebrionis*, called “Col-Kill”, demonstrated efficacy in the control of the coleopteran *Phaedon brassicae* [141]; (ii) for the control of lepidopteran *Plutella xylostella* and *Spodoptera exigua*, the mixture called “Dual Bt-Plus” by *B. thuringiensis kurstaki* and *B. thuringiensis aizawai* with culture broth *X. nematophila* [155]; and (iii) some authors described the ability of *Xenorhabdus* and *Photorhabdus* bacteria to increase the toxic effect of Cry4Ba derived from *Bti* against *Ae. aegypti* [29, 64]. In addition, mixtures of *X. nematophila* or *P. temperata temperata* were tested to verify the suppression effect of immune responses on insects and consequently increased toxicity of *B. thuringiensis* [44]. These authors also hypothesized that some metabolites of *Xenorhabdus* and/or *Photorhabdus* could be related to inhibition of eicosanoid synthesis and increased *Bti* toxicity against mosquitoes. Thus, they used a mixture of *Bti* spores with *X. nematophila* culture broth containing metabolites. This solution showed greater efficacy in the control of *Ae. albopictus* mosquitoes and *Cx. pipiens pallens*, with an increase in *Bti* toxicity against these insect species. Based on these results, they developed an insecticide called “Dip-Kill” [44]. The culture broths of *Xenorhabdus hominickii* and *P. temperata temperata* were also able to increase the toxicity of *Bti* against culicides [44].

The toxic effect of a bacterial cultures of *X. nematophila* and *P. luminescens*, was also tested in *Ae. aegypti* by Silva et al. [13]. Both culture broths of *X. nematophila* and *P. luminescens*, caused larvae mortality, and interfered the development of pupae and adults. These authors observed greater larvicidal stability of *X. nematophila* culture fluids exposed to high temperatures (100 °C) in contrast to *P. luminescens* culture fluids tested. The temperature labile pathogenicity of *Photorhabdus* bacteria may be related to both proteins and secondary metabolites that are relatively unstable [13]. However, bioactive compounds produced by *Xenorhabdus* are stable and therefore potential agents for a putative application in mosquito control [14].

The bacterium *X. innexi*, when injected into several species of insects, does not show insect pathogenicity, but when using cultures fluids of cells, some isolates presented larvicide activities against *Aedes*, *Culex* and *Anopheles*. The Xlt compound, derived from this bacterial species, has been described as a low molecular weight lipopeptide that has toxic activity against mosquito larvae. The protein composition has a high content of amino acids such as histidine, glycine, asparagine/aspartate, diaminobutyric acid and serine. The lipid portion has at least one oxo-fatty acid (C8 - C20) [150]. Thus, Kim et al. [118] analyzed Xlt’s specificity and mechanism of action against mosquito larvae. Different doses were used for exposure of *Ae. aegypti* larvae, *Cx. pipiens* and *An. gambiae*. The authors observed that Xlt is mainly toxic to mosquito larvae, considering that pupae and adults were not affected in pathogenicity bioassays. The effects of Xlt were also observed by Kim et al. [118] in different cell strains of insects, including *Ae. aegypti* (Aag-2), *D. melanogaster* (S2) and *M. sexta* (GV1). After treatment for six hours (CL_50_ for mosquito larvae), no alterations in the cellular morphology of lepidopteran were observed. However, *Ae. aegypti* cells (Aag2) presented aggregation followed by induced apoptosis after the same exposure time. In the cell viability analysis (using SYTOX®), it was possible to observe that only mosquito cells emit fluorescence, indicating that Xlt at low doses has no toxic effects on non-target insect cells.

The Xlt toxicity was also evaluated in fibroblasts (Hs68) and mast cells (HMC-1), compared to *Ae. aegypti* strains (Aag-2) and *Ae. albopictus* (C6/36), and in 24 hours of treatment (1 ppm), more than 80% of mosquito cells were killed. On the other hand, human cells were not affected at the same dose. Only with the dose of 50 ppm (significantly higher) of Xlt, the Hs68 fibroblast strain showed changes in cellular viability, with a decreased number of cells, but compared to mosquito cells Aag-2 and C6/36, the Hs68 strain presented greater viability after treatment with doses of 50 and 100 ppm. However, the HMC-1 human mast cell population strain showed an increase in the cell population after exposure to 10 and 50 ppm of Xlt. According to the authors, the increase in the number of HMC-1 mast cells may have occurred due to the stimulation of peptides or lipoproteins that induce the activation of these cells that are components of the immune system [156]. Thus, demonstrating that Xlt of *X. innexi* it is more toxic to mosquito cells in comparison to human cell strains [118].

Due to the existence of Xlt toxicity after ingestion by the larvae, it is possible that the mechanism of action could be similar to *Bti* toxins that act in the midgut of insects [157]. However, Kim et al. [118] suggested that Xlt has the ability to create pores on the apical surface of cells in the anterior midgut of mosquitoes but in the anterior portion of the middle intestine, causing cell death. In *Ae. aegypti* larvae at the beginning of the fourth stage, after exposure to Xlt, the pH of the anterior midgut became more acidic. According to Boudko et al. [158], the rupture of intestinal integrity causes a decrease in the pH. Although both *Bti* and Xlt act in the intestines of the larvae, it is worth mentioning that there are probably specific cell connection sites, spatially altering the place of action of biological agents against mosquito larvae [118], as it has been demonstrated that *Bti* acts at the posterior portion of the middle intestine [159, 160] (Fig. 3).

Other bacterial isolates were also tested for toxicity to insects that did not present significant effects on mortality. Some of these are listed here: (i) *Xenorhabdus stockiae* (bLPA12.2_TH, bCR7.3_TH and bPH23.5_TH), *Xenorhabdus miraniensis* (bMH16.4_TH, bMH16.1_TH and bMH4.5_TH) and *Photorhabdus* (bPY17.4_TH, bLPO16.2_TH, bMH8.4_TH and bNA22.1_TH) were not effective in the mortality of *Ae. aegypti* larvae [27]; and (ii) *Xenorhabdus japonica* (bNN165.4_TH) and *Xenorhabdus vietnamensis* (bNN167.2_TH and bNN167.3_TH) presented low toxicity to *Ae. aegypti* and *Ae. albopictus* larvae, probably due to the absence of secretion of toxic metabolites for the species of tested culicids [14]. Other factors may be related to the mortality or survival of insects, such as the number of bacterial cells ingested, production of metabolites in water and, difference in compounds secreted by different bacterial strains [14].

Recently Kajla et al. [161] described *Xenorhabdus budapestensis* fabclavines activities with mosquito-repellent action. The authors found that compounds of *X. budapestensis* cultures are capable of inhibiting artificial hematophagy of females of *Aedes*, *Anopheles* and *Culex*, probably due to the presence of fabclavines. They argue that possibly amino acids asparagine/aspartate and histidine, perhaps 2,3-diaminobutyric acid, would be related to the repellent effect of bacteria to mosquitoes [73]. It has been shown that this repellent activity of compounds secreted by *X. budapestensis* may be superior to the repellents commonly used against *Ae. aegypti*, such as DEET or picaridin [161]. The authors suggested that these bioactive compounds of *Xenorhabdus* and *Photorhabdus* may exercise larvicidal action against mosquitoes when used in breeding sites, because they are used as a food source of the larvae.

## Perspectives on applicability in mosquito control

Bacteria of the genera *Photorhabdus* and *Xenorhabdus* stand out for being very effective to control *Ae. aegypti* after oral uptake by the mosquito larvae. *Photorhabdus luminescens* and *X. nematophila* were used as a food source for *Ae. aegypti* larvae. After 24 hours 50% of larvae were dead, culminating to 100% in 96 hours [29]. However, by exposing *Ae. aegypti* larvae to a series dilution of *P. luminescens* and *X. nematophila* crude culture fluids diluted in distilled water, 100% mortality was observed after 4 hours for both bacteria species. So, it seems that crude culture fluids of both *Photorhabdus* and *Xenorhabdus* are highly effective in a short period of time after oral intake to obtain mortality against larvae of *Ae. aegypti*, compared to other pathogenic bacteria for mosquitoes. For example, vegetative cells of *B. thuringiensis* need at least 12 hours to kill *Ae. aegypti* larvae [162] or, the strains of *B. thuringiensis* (SV2) and *Serratia* sp. (SV6), which only reach mortality of 50% after six and 12 hours of exposure, respectively, in larvae of *Ae. aegypti*, *Anopheles stephensi* and *Cx. quinquefasciatus* [163].

Fukruksa et al. [27] also noted advantages of the use of *Xenorhabdus* and *Photorhabdus* due to the rapid capacity of mortality range of larvae of *Aedes* spp. *Xenorhabdus ehlersii* (bMH9.2_TH) presented greater effectiveness against both fed and non-fed *Ae. aegypti* larvae, with a range of 100% mortality in up to 96 hours. On the other hand, for larvae of the same mosquito species, the isolate of *Xenorhabdus stockiae* (bLPA18.4_TH) has a mortality rate greater than 60% in 72 and 96 hours. The authors highlight the potential of isolate *X. ehlersii* bMH9.2_TH as more pathogenic, opening possibilities for *X. ehlersii* to be a biological control agent for *Ae. aegypti*.

The isolate (bNN112.3_TH) from *X. stockiae* has been tested for exposure of *Ae. aegypti* larvae, in which the authors observed 99% mortality after 96 hours. Another bioassay, with *Ae. albopictus*, demonstrated mortality of 98% of larvae after exposure of 96 hours to *P. luminescens akhurstii* (bNN121.4_TH). The authors highlight the potential of these isolates as control agents against the two species of mosquitoes to the bacteria they were exposed to [14].

The evidence that *Xenorhabdus* and *Photorhabdus* bacteria synthetize a diversity of secondary metabolites opens possibilities for these compounds to be even more specific and potent agents of biological control of mosquitoes. For example, the chemical change in the structure of fabclavines or their use in combination with chemical or biological insecticides is already established. For the use of these metabolites in the control of culicids, toxicity to other insects, aquatic organisms and humans has to be evaluated. Field applicability and feasibility of large-scale production also has to be analyzed in the future [26].

In addition, research aimed at the isolation, identification and characterization of bioactive compounds is of vital importance for elucidating the mechanisms of action of secondary toxins/metabolites that are responsible for the death of *Ae. aegypti* larvae [13, 29]. However, the molecular mechanisms of action were not elucidated. PirAB proteins are larvicide potentials for the control of vector mosquitoes, and it is necessary to conduct studies on biosafety aspects for the use of these proteins [59]. Finally, it is necessary to understand the application of these bioactive compounds to be implanted in the biological control of mosquitoes [27]. Therefore, it is necessary to study the period of activity of these entomopathogenic bacteria and their toxic compounds, as well as the time of their residual effect on mosquito breeding sites.

## Conclusions

The current methods of controlling these mosquitoes, which are indicated by World Health Organization, have shown problems as a high cost for biological and chemical control. They often have low specificity for the target organisms and are therefore also toxic to non-target organisms, including humans. In addition, recent studies reported resistance to the most commonly used insecticides, such as the organophosphate temephos and pyrethroids in several populations of *Ae. aegypti* and *Ae. albopictus* distributed worldwide. Thus, the control of these insects depends on a wide variety of chemical and biological arsenals that can contribute to the prevention of their control. Entomopathogenic bacteria such as *Photorhabdus* and *Xenorhabdus* should be considered in these arsenals, since so many researchers have demonstrated their efficiency against mosquitoes. Therefore, this observation opens possibilities for more insect specific compounds and potent agents of biological control of mosquitoes. Entomopathogenic bacteria have to be urgently considered for mosquito control in the near future.

## Data Availability

Not applicable.

## Abbreviations

Aag-2: Cell strain of *Aedes aegypti*
*Bti*: *Bacillus thuringiensis israelensis*
C6/36: Cell strain of *Aedes albopictus*
CFU’s: Colony-forming units
EPNs: Entomopathogenic nematodes
GV1: Cell strain of *Manduca sexta*
HMC-1: Human mast cell lines
Hs68: Human fibroblasts cell lines
Mcf: Make caterpillars floppy
pH: Power of hydrogen
Pir: Insect-related protein
PO: Phenoloxidase
Ppm: Parts per million
*Pr*: *Photorhabdus*
*Pra*: *Photorhabdus asymbiotica*
proPO: Prophenoloxidase
Pvc: *Photorhabdus* virulence cassettes
S2: Cell strain of *Drosophila melanogaster*
SV2: Cell strain of *Bacillus thuringiensis*
SV6: Cell strain of *Serratia* sp
TccC3 and TccC5: *Photorhabdus luminescens* toxins
Tcs: Toxin complexes
Xlt: *Xenorhabdus* lipoprotein toxin
*Xr*: *Xenorhabdus*
*Xrn*: *Xenorhabdus nematophila*

## References

[1] Nuckols JT, Huang YJS, Higgs S, Miller AL, Pyles RB, Spratt HM (2015). Evaluation of simultaneous transmission of chikungunya virus and dengue virus type 2 in infected *Aedes aegypti* and *Aedes albopictus* (Diptera: Culicidae). J Med Entomol..

[2] Gratz NG (1999). Emerging and resurging diseases. CRC Handb Mar Mammal Med..

[3] Benelli G, Mehlhorn H (2016). Declining malaria, rising of dengue and Zika virus: insights for mosquito vector control. Parasitol Res..

[4] Lahariya C, Pradhan SK (2006). Emergence of chikungunya virus in Indian subcontinent after 32 years: a review. J Vector Borne Dis..

[5] Nunes MRT, Faria NR, de Vasconcelos JM, Golding N, Kraemer MUG, de Oliveira LF (2015). Emergence and potential for spread of chikungunya virus in Brazil. BMC Med..

[6] da Silva NM, Teixeira RAG, Cardoso CG, Siqueira Junior JB, Coelho GE, de Oliveira ESF (2018). Vigilância de chikungunya no Brasil: desafios no contexto da Saúde Pública. Epidemiol e Serv saude Rev do Sist Unico Saude do Bras..

[7] Smithburn KC, Hughes TP, Burke AW, Paul JH (1940). A neurotropic virus isolated from the blood of a native of Uganda 1. Am J Trop Med Hyg..

[8] Petersen LR, Hayes EB (2008). West Nile virus in the Americas. Med Clin North Am..

[9] Kilpatrick AM (2011). Globalization, land use and the invasion of West Nile virus. Science..

[10] Malone RW, Homan J, Callahan MV, Glasspool-Malone J, Damodaran L, Schneider ADB (2016). Zika virus: medical countermeasure development challenges. PLoS Negl Trop Dis..

[11] Vorou R (2016). Zika virus, vectors, reservoirs, amplifying hosts, and their potential to spread worldwide: what we know and what we should investigate urgently. Int J Infect Dis..

[12] Lemine MMA, Ould Lemrabott MA, Hasni Ebou M, Mint Lekweiry K, Ould Ahmedou Salem MS, Ould Brahim K (2017). Mosquitoes (Diptera: Culicidae) in Mauritania: a review of their biodiversity, distribution and medical importance. Parasit Vectors..

[13] da Silva JLR, Schwalm FU, Silva CE, da Costa M, Heermann R, da Silva OS (2017). Larvicidal and growth-Inhibitory activity of entomopathogenic bacteria culture fluids against *Aedes aegypti* (Diptera: Culicidae). J Econ Entomol..

[14] Yooyangket T, Muangpat P, Polseela R, Tandhavanant S, Thanwisai A, Vitta A (2018). Identification of entomopathogenic nematodes and symbiotic bacteria from Nam Nao National Park in Thailand and larvicidal activity of symbiotic bacteria against *Aedes aegypti* and *Aedes albopictus*. PLoS ONE..

[15] Kumar PM, Kovendan K, Murugan K (2013). Integration of botanical and bacterial insecticide against *Aedes aegypti* and *Anopheles stephensi*. Parasitol Res..

[16] Bhatt S, Gething PW, Brady OJ, Messina JP, Farlow AW, Moyes CL (2013). The global distribution and burden of dengue. Nature..

[17] Villabona-Arenas CJ, de Zanotto PMA (2013). Worldwide spread of dengue virus type 1. PLoS One..

[18] Kraemer MUG, Sinka ME, Duda KA, Mylne AQN, Shearer FM, Barker CM (2015). The global distribution of the arbovirus vectors *Aedes aegypti* and *Ae. albopictus*. Elife..

[19] Laughlin CA, Morens DM, Cassetti MC, Costero-Saint Denis A, San Martin JL, Whitehead SS (2012). Dengue research opportunities in the Americas. J Infect Dis..

[20] Scott TW, Takken W (2012). Feeding strategies of anthropophilic mosquitoes result in increased risk of pathogen transmission. Trends Parasitol..

[21] Soo KM, Khalid B, Ching SM, Chee HY (2016). Meta-analysis of dengue severity during infection by different dengue virus serotypes in primary and secondary infections. PLoS ONE ..

[22] Vythilingam I, Sam JIC, Chan YF, Khaw LT, Wan Sulaiman WY (2016). New paradigms for virus detection, surveillance and control of Zika virus vectors in the settings of Southeast Asia. Front Microbiol..

[23] Guha L, Seenivasagan T, Bandyopadhyay P, Thanvir Iqbal S, Sathe M, Sharma P (2012). Oviposition and flight orientation response of *Aedes aegypti* to certain aromatic aryl hydrazono esters. Parasitol Res..

[24] Tauil PL (2002). Critical aspects of dengue control in Brazil. Cad Saude Publica..

[25] Rochlin I, Ninivaggi DV, Hutchinson ML, Farajollahi A (2013). Climate change and range expansion of the Asian tiger mosquito (*Aedes albopictus*) in northeastern USA: implications for public health practitioners. PLoS ONE..

[26] Kajla MK (2019). Symbiotic bacteria as potential agents for mosquito control. Trends Parasitol..

[27] Fukruksa C, Yimthin T, Suwannaroj M, Muangpat P, Tandhavanant S, Thanwisai A (2017). Isolation and identification of *Xenorhabdus* and *Photorhabdus* bacteria associated with entomopathogenic nematodes and their larvicidal activity against *Aedes aegypti*. Parasit Vectors..

[28] Gubler DJ (2011). Emerging vector-borne flavivirus diseases: are vaccines the solution?. Expert Rev Vaccines..

[29] Da Silva OS, Prado GR, Da Silva JLR, Silva CE, Da Costa M, Heermann R (2013). Oral toxicity of *Photorhabdus luminescens* and *Xenorhabdus nematophila* (Enterobacteriaceae) against *Aedes aegypti* (Diptera: Culicidae). Parasitol Res..

[30] Baldacchino F, Caputo B, Chandre F, Drago A, della Torre A, Montarsi F (2015). Control methods against invasive *Aedes* mosquitoes in Europe: a review. Pest Manag Sci..

[31] Bellini R, Medici A, Puggioli A, Balestrino F, Carrieri M (2013). Pilot Field Trials With *Aedes albopictus* irradiated sterile males in Italian urban areas. J Med Entomol..

[32] Winskill P, Harris AF, Morgan SA, Stevenson J, Raduan N, Alphey L (2014). Genetic control of *Aedes aegypti*: data-driven modelling to assess the effect of releasing different life stages and the potential for long-term suppression. Parasit Vectors..

[33] Da Silva JJ, Mendes J (2007). Susceptibility of *Aedes aegypti* (L.) to the insect growth regulators diflubenzuron and methoprene in Uberlândia, State of Minas Gerais. Rev Soc Bras Med Trop..

[34] Lucia A, Harburguer L, Licastro S, Zerba E, Masuh H (2009). Efficacy of a new combined larvicidal-adulticidal ultralow volume formulation against *Aedes aegypti* (Diptera: Culicidae), vector of dengue. Parasitol Res..

[35] Salokhe SG, Deshpande SG, Mukherjee SN (2012). Evaluation of the insect growth regulator Lufenuron (Match®) for control of *Aedes aegypti* by simulated field trials. Parasitol Res..

[36] Mazzarri MB, Georghiou GP (1995). Characterization of resistance to organophosphate, carbamate, and pyrethroid insecticides in field populations of *Aedes aegypti* from Venezuela. J Am Mosq Control Assoc..

[37] Braga IA, Mello CB, Montella IR, Lima JBP, Júnior ADJM, Medeiros PFV (2005). Effectiveness of methoprene, an insect growth regulator, against temephos-resistant *Aedes aegypti* populations from different Brazilian localities, under laboratory conditions. J Med Entomol..

[38] Naqqash MN, Gökçe A, Bakhsh A, Salim M (2016). Insecticide resistance and its molecular basis in urban insect pests. Parasitol Res..

[39] Smith TM, Stratton GW (1986). Effects of synthetic pyrethroid insecticides on nontarget organisms. Residue Rev..

[40] Casida JE, Quistad GB (1998). Golden age of insecticide research: past, present, or future?. Annu Rev Entomol..

[41] Hemingway J, Ranson H (2000). Insecticide resistance in insect vectors of human disease. Annu Rev Entomol..

[42] Hao L, Johnson K, Cursino L, Mowery P, Burr TJ (2017). Characterization of the *Xylella fastidiosa* PD1311 gene mutant and its suppression of Pierce’s disease on grapevines. Mol Plant Pathol..

[43] Kim IH, Aryal SK, Aghai DT, Casanova-Torres ÁM, Hillman K, Kozuch MP (2017). The insect pathogenic bacterium *Xenorhabdus innexi* has attenuated virulence in multiple insect model hosts yet encodes a potent mosquitocidal toxin. BMC Genomics..

[44] Park Y, Kyo Jung J, Kim Y (2016). A mixture of *Bacillus thuringiensis* subsp. *israelensis* with *Xenorhabdus nematophila*-cultured broth enhances toxicity against mosquitoes *Aedes albopictus* and *Culex pipiens pallens* (Diptera: Culicidae). J Econ Entomol..

[45] Merritt R (1992). Feeding behavior, natural food, and nutritional relationships of larval mosquitos. Annu Rev Entomol..

[46] Hoffmann AA, Montgomery BL, Popovici J, Iturbe-Ormaetxe I, Johnson PH, Muzzi F (2011). Successful establishment of *Wolbachia* in *Aedes* populations to suppress dengue transmission. Nature..

[47] Lu P, Bian G, Pan X, Xi Z (2012). *Wolbachia* induces density-dependent inhibition to dengue virus in mosquito cells. PLoS Negl Trop Dis..

[48] Otta DA, Rott MB, Carlesso AM, Da Silva OS (2012). Prevalence of *Acanthamoeba* spp. (Sarcomastigophora: Acanthamoebidae) in wild populations of *Aedes aegypti* (Diptera: Culicidae). Parasitol Res..

[49] Leles RN, D’Alessandro WB, Luz C (2012). Effects of *Metarhizium anisopliae* conidia mixed with soil against the eggs of *Aedes aegypti*. Parasitol Res..

[50] Kovendan K, Murugan K, Vincent S, Kamalakannan S (2011). Larvicidal efficacy of *Jatropha curcas* and bacterial insecticide, *Bacillus thuringiensis*, against lymphatic filarial vector, *Culex quinquefasciatus* Say (Diptera: Culicidae). Parasitol Res..

[51] Mardini LB, Souza MA, Rabinovitch L, Alves RS, Silva CM (1999). Field studies with the bacterial larvicide INPALBAC for *Simulium* spp. control in Rio Grande do Sul. Brazil. Mem Inst Oswaldo Cruz..

[52] Rabinovitch L, Cavados CF, Chaves JQ, Coutinho CJ, Zahner V, Silva KR (1999). A new strain of *Bacillus thuringiensis* serovar *israelensis* very active against blackfly larvae. Mem Inst Oswaldo Cruz..

[53] De Araújo-Coutinho CJPDC, Cunha ADBPV, Serra-Freire NM, De Mello RP. Evaluation of the impact of Bacillus thuringiensis serovar israelensis and temephos, used for the control of Simulium (Chirostilbia) pertinax Kollar (1832). (Diptera, Simuliidae) on the associated entomofauna, Paraty, State of Rio de Janeiro, Brazil. Mem Inst Oswaldo Cruz..

[54] Bravo A, Likitvivatanavong S, Gill SS, Soberon M (2011). *Bacillus thuringiensis*: a story of a successful bioinsecticide. Insect Biochem Mol Biol..

[55] Lacey LA (2007). *Bacillus thuringiensis* serovariety *israelensis* and *Bacillus sphaericus* for Mosquito Control. J Am Mosq Control Assoc..

[56] Lacey LA, Grzywacz D, Shapiro-Ilan DI, Frutos R, Brownbridge M, Goettel MS (2015). Insect pathogens as biological control agents: back to the future. J Invertebr Pathol..

[57] Geissbühler Y, Kannady K, Chaki PP, Emidi B, Govella NJ, Mayagaya V (2009). Microbial larvicide application by a large-scale, community-based program reduces malaria infection prevalence in urban Dar Es Salaam, Tanzania. PLoS ONE..

[58] Chattopadhyay A, Bhatnagar NB, Bhatnagar R (2004). Bacterial insecticidal toxins. Crit Rev Microbiol..

[59] Ahantarig A, Chantawat N, Waterfield NR, Ffrench-Constant R, Kittayapong P (2009). PirAB toxin from *Photorhabdus asymbiotica* as a larvicide against dengue vectors. Appl Environ Microbiol..

[60] Gomes SA, Paula AR, Ribeiro A, Moraes COP, Santos JWAB, Silva CP (2015). Neem oil increases the efficiency of the entomopathogenic fungus *Metarhizium anisopliae* for the control of *Aedes aegypti* (Diptera: Culicidae) larvae. Parasit Vectors..

[61] Jaber S, Mercier A, Knio K, Brun S, Kambris Z (2016). Isolation of fungi from dead arthropods and identification of a new mosquito natural pathogen. Parasit Vectors..

[62] Veronesi R, Carrieri M, Maccagnani B, Maini S, Bellini R (2015). *Macrocyclops albidus* (Copepoda: Cyclopidae) for the Biocontrol of *Aedes albopictus* and *Culex pipiens* in Italy. J Am Mosq Control Assoc..

[63] Kovendan K, Murugan K, Vincent S, Barnard DR (2012). Studies on larvicidal and pupicidal activity of *Leucas aspera* Willd. (Lamiaceae) and bacterial insecticide, *Bacillus sphaericus*, against malarial vector, *Anopheles stephensi* Liston. (Diptera: Culicidae). Parasitol Res..

[64] Park Y (2015). Entomopathogenic bacterium, *Xenorhabdus nematophila* and *Photorhabdus luminescens*, enhances *Bacillus thuringiensis* Cry4Ba toxicity against yellow fever mosquito, *Aedes aegypti* (Diptera: Culicidae). J Asia Pac Entomol..

[65] Setha T, Chantha N, Benjamin S, Socheat D (2016). Bacterial larvicide, *Bacillus thuringiensis israelensis* strain AM 65-52 water dispersible granule formulation impacts both dengue vector, *Aedes aegypti* (L.) population density and disease transmission in Cambodia. PLoS Negl Trop Dis..

[66] Bowen DJ, Ensign JC (1998). Purification and characterization of a high-molecular-weight insecticidal protein complex produced by the entomopathogenic bacterium *Photorhabdus luminescens*. Appl Environ Microbiol..

[67] Sergeant M, Jarrett P, Ousley M, Morgan JAW (2003). Interactions of insecticidal toxin gene products from *Xenorhabdus* nematophilus PMFI296. Appl Environ Microbiol..

[68] Pidot SJ, Coyne S, Kloss F, Hertweck C (2014). Antibiotics from neglected bacterial sources. Int J Med Microbiol..

[69] Bode HB (2009). Entomopathogenic bacteria as a source of secondary metabolites. Curr Opin Chem Biol..

[70] Hinchliffe SJ (2013). Insecticidal toxins from the *Photorhabdus* and *Xenorhabdus* bacteria. Open Toxinol J..

[71] Tobias NJ, Wolff H, Djahanschiri B, Grundmann F, Kronenwerth M, Shi YM (2017). Natural product diversity associated with the nematode symbionts *Photorhabdus* and *Xenorhabdus*. Nat Microbiol..

[72] Furgani G, Böszörményi E, Fodor A, Máthé-Fodor A, Forst S, Hogan JS (2008). *Xenorhabdus* antibiotics: a comparative analysis and potential utility for controlling mastitis caused by bacteria. J Appl Microbiol..

[73] Fuchs SW, Grundmann F, Kurz M, Kaiser M, Bode HB (2014). Fabclavines: bioactive peptide-polyketide-polyamino hybrids from *Xenorhabdus*. ChemBioChem..

[74] Masschelein J, Clauwers C, Stalmans K, Nuyts K, De Borggraeve W, Briers Y (2015). The zeamine antibiotics affect the integrity of bacterial membranes. Appl Environ Microbiol..

[75] Thanwisai A, Tandhavanant S, Saiprom N, Waterfield NR, Ke Long P, Bode HB (2012). Diversity of *Xenorhabdus* and *Photorhabdus* spp. and their symbiotic entomopathogenic nematodes from Thailand. PLoS ONE..

[76] Grundmann F, Kaiser M, Schiell M, Batzer A, Kurz M, Thanwisai A (2014). Antiparasitic chaiyaphumines from entomopathogenic *Xenorhabdus* sp. PB61.4. J Nat Prod..

[77] Antonello AM, Sartori T, Folmer Correa AP, Brandelli A, Heermann R, Rodrigues Júnior LC (2017). Entomopathogenic bacteria *Photorhabdus luminescens* as drug source against *Leishmania amazonensis*. Parasitology..

[78] Antonello AM, Sartori T, Silva MB, Prophiro JS, Pinge-Filho P, Heermann R (2019). Anti-*Trypanosoma* activity of bioactive metabolites from *Photorhabdus luminescens* and *Xenorhabdus nematophila*. Exp Parasitol..

[79] Ruiu L, Satta A, Floris I (2013). Emerging entomopathogenic bacteria for insect pest management. Bull Insectology..

[80] Daborn PJ, Waterfield N, Silva CP, Au CPY, Sharma S, Ffrench-Constant RH (2002). A single *Photorhabdus* gene, makes caterpillars floppy (mcf), allows *Escherichia coli* to persist within and kill insects. Fixed Point Theory Appl..

[81] Benfarhat TD, Amira AB, Khedher SB, Givaudan A, Jaoua S, Tounsi S (2013). Combinatorial effect of *Bacillus thuringiensis* kurstaki and *Photorhabdus luminescens* against *Spodoptera littoralis* (Lepidoptera: Noctuidae). J Basic Microbiol..

[82] Shi H, Zeng H, Yang X, Zhao J, Chen M, Qiu D (2012). An insecticidal protein from *Xenorhabdus ehlersii* triggers prophenoloxidase activation and hemocyte decrease in *Galleria mellonella*. Curr Microbiol..

[83] Shi HX, Zeng HM, Yang XF, Liu Z, Qiu D (2013). An insecticidal protein from *Xenorhabdus ehlersii* stimulates the innate immune response in *Galleria mellonella*. World J Microbiol Biotechnol..

[84] Bussaman P, Sa-Uth C, Rattanasena P, Chandrapatya A (2012). Acaricidal activities of whole cell suspension, cell-free supernatant, and crude cell extract of *Xenorhabdus stokiae* against mushroom mite (*Luciaphorus* sp). J Zhejiang Univ Sci B..

[85] Bussaman P, Rattanasena P (2016). Additional property of *Xenorhabdus stockiae* for inhibiting cow mastitis-causing bacteria. Biosci Biotechnol Res Asia..

[86] Namsena P, Bussaman P, Rattanasena P (2016). Bioformulation of *Xenorhabdus stockiae* PB09 for controlling mushroom mite, *Luciaphorus perniciosus* Rack. Bioresour Bioprocess..

[87] Zhou X, Kaya HK, Heungens K, Goodrich-Blair H (2002). Response of ants to a deterrent factor(s) produced by the symbiotic bacteria of entomopathogenic nematodes. Appl Environ Microbiol..

[88] Gulcu B, Hazir S, Kaya HK (2012). Scavenger deterrent factor (SDF) from symbiotic bacteria of entomopathogenic nematodes. J Invertebr Pathol..

[89] Grimont PAD, Steigerwalt AG, Boemare N (1984). Deoxyribonucleic acid relatedness and phenotypic study of the genus *Xenorhabdus*. Int J Syst Bacteriol..

[90] Boemare NE, Akhurst RJ, Mourant RG (1993). DNA relatedness between *Xenorhabdus* spp. (Enterobacteriaceae), symbiotic bacteria of entomopathogenic nematodes, and a proposal to transfer *Xenorhabdus luminescens* to a new genus, *Photorhabdus* gen. nov. Int J Syst Bacteriol..

[91] Fischer-Le Saux M, Viallard V, Brunel B, Normand P, Boemare NE (1999). Polyphasic classification of the genus *Photorhabdus* and proposal of new taxa: *P luminescens* subsp. *luminescens* subsp. nov., *P. luminescens* subsp. *akhurstii* subsp. nov., *P. luminescens* subsp. *laumondii* subsp. nov., *P. temperata* sp. nov., *P. temperata* subs. *temperata* subsp. nov., and *P. asymbiotica* sp. nov. Int J Syst Bacteriol..

[92] Brillard J, Ribeiro C, Boemare N, Brehélin M, Givaudan A (2001). Two distinct hemolytic activities in *Xenorhabdus nematophila* are active against immunocompetent insect cells. Appl Environ Microbiol..

[93] Tailliez P, Pagès S, Ginibre N, Boemare N (2006). New insight into diversity in the genus *Xenorhabdus*, including the description of ten novel species. Int J Syst Evol Microbiol..

[94] Maneesakorn P, An R, Daneshvar H, Taylor K, Bai X, Adams BJ (2011). Phylogenetic and cophylogenetic relationships of entomopathogenic nematodes (*Heterorhabditis*: Rhabditida) and their symbiotic bacteria (*Photorhabdus*: Enterobacteriaceae). Mol Phylogenet Evol..

[95] Glaeser SP, Tobias NJ, Thanwisai A, Chantratita N, Bode HB (2017). Kämpfer P *Photorhabdus luminescens* subsp. *namnaonensis* subsp. nov., isolated from *Heterorhabditis baujardi* nematodes. Int J Syst Evol Microbiol..

[96] Muangpat P, Yooyangket T, Fukruksa C, Suwannaroj M, Yimthin T, Sitthisak S (2017). Screening of the antimicrobial activity against drug resistant bacteria of *Photorhabdus* and *Xenorhabdus* associated with entomopathogenic nematodes from Mae Wong National Park, Thailand. Front Microbiol..

[97] Thomas GM, Poinar GO (1979). *Xenorhabdus* gen. nov., a genus of entomopathogenic, nematophilic bacteria of the family Enterobacteriacease. Int J Syst Bacteriol..

[98] Akhurst RJ (1980). Morphological and functional dimorphism in *Xenorhabdus* spp. bacteria symbiotically associated with the insect pathogenic nematodes *Neoaplectana* and *Heterorhabditis*. Microbiology..

[99] Forst S, Dowds B, Boemare N, Stackebrandt E (1997). *Xenorhabdus* and *Photorhabdus* spp. bugs that kill bugs. Annu Rev Microbiol..

[100] Gerrard JG, Joyce SA, Clarke DJ, Ffrench-Constant RH, Nimmo GR, Looke DFM (2006). Nematode symbiont for *Photorhabdus asymbiotica*. Emerg Infect Dis..

[101] Goodrich-Blair H, Clarke DJ (2007). Mutualism and pathogenesis in *Xenorhabdus* and *Photorhabdus*: two roads to the same destination. Mol Microbiol..

[102] Bird AF, Akhurst RJ (1983). The nature of the intestinal vesicle in nematodes of the family steinernematidae. Int J Parasitol..

[103] Kim SK, Flores-Lara Y, Patricia Stock S (2012). Morphology and ultrastructure of the bacterial receptacle in *Steinernema* nematodes (Nematoda: Steinernematidae). J Invertebr Pathol..

[104] Chaston JM, Suen G, Tucker SL, Andersen AW, Bhasin A, Bode E (2011). The entomopathogenic bacterial endosymbionts *Xenorhabdus* and *Photorhabdus*: convergent lifestyles from divergent genomes. PLoS ONE.

[105] Dillman AR, Guillermin ML, Lee JH, Kim B, Sternberg PW, Hallem EA (2012). Olfaction shapes host-parasite interactions in parasitic nematodes. Proc Natl Acad Sci USA.

[106] Kaya HK, Gaugler R (1993). Entomopathogenic nematodes. Annu Rev Entomol..

[107] Gulcu B, Cimen H, Raja RK, Hazir S (2017). Entomopathogenic nematodes and their mutualistic bacteria: their ecology and application as microbial control agents. Biopestic Int..

[108] Park Y, Kim Y (2000). Eicosanoids rescue *Spodoptera exigua* infected with *Xenorhabdus nematophilus*, the symbiotic bacteria to the entomopathogenic nematode *Steinernema carpocapsae*. J Insect Physiol..

[109] Eom S, Park Y, Kim Y (2014). Sequential immunosuppressive activities of bacterial secondary metabolites from the entomopahogenic bacterium *Xenorhabdus nematophila*. J Microbiol..

[110] Ffrench-Constant RH, Dowling A, Waterfield NR (2007). Insecticidal toxins from *Photorhabdus* bacteria and their potential use in agriculture. Toxicon..

[111] Herbert EE, Goodrich-Blair H (2007). Friend and foe: the two faces of X*enorhabdus nematophila*. Nat Rev Microbiol..

[112] Park Y, Kim Y, Yi Y (1999). Identification and characterization of a symbiotic bacterium associated with *Steinernema carpocapsae* in Korea. J Asia Pac Entomol..

[113] Kang S, Han S, Kim Y (2004). Identification of an entomopathogenic bacterium, *Photorhabdus temperata* subsp. *temperata*, in Korea. J Asia Pac Entomol..

[114] Sicard M, Le Brun N, Pages S, Godelle B, Boemare N, Moulia C (2003). Effect of native *Xenorhabdus* on the fitness of their *Steinernema* hosts: contrasting types of interaction. Parasitol Res..

[115] Veesenmeyer JL, Andersen AW, Lu X, Hussa EA, Murfin KE, Chaston JM (2014). NilD CRISPR RNA contributes to *Xenorhabdus nematophila* colonization of symbiotic host nematodes. Mol Microbiol..

[116] Weissfeld AS, Halliday RJ, Simmons DE, Trevino EA, Vance PH, O’Hara CM (2005). *Photorhabdus asymbiotica*, a pathogen emerging on two continents that proves that there is no substitute for a well-trained clinical microbiologist. J Clin Microbiol..

[117] Owuama CI (2001). Entomopathogenic symbiotic bacteria, *Xenorhabdus* and *Photorhabdus* of nematodes. World J Microbiol Biotechnol..

[118] Kim IH, Ensign J, Kim DY, Jung HY, Kim NR, Choi BH (2017). Specificity and putative mode of action of a mosquito larvicidal toxin from the bacterium *Xenorhabdus innexi*. J Invertebr Pathol..

[119] Zhang H, Mao J, Liu F, Zeng F (2012). Expression of a nematode symbiotic bacterium-derived protease inhibitor protein in tobacco enhanced tolerance against *Myzus persicae*. Plant Cell Rep..

[120] Kumari P, Mahapatro GK, Banerjee N, Sarin NB (2015). Ectopic expression of GroEL from *Xenorhabdus nematophila* in tomato enhances resistance against *Helicoverpa armigera* and salt and thermal stress. Transgenic Res..

[121] Liu D, Burton S, Glancy T, Li ZS, Hampton R, Meade T (2003). Insect resistance conferred by 283-kDa *Photorhabdus luminescens* protein TcdA in *Arabidopsis thaliana*. Nat Biotechnol..

[122] Boemare NE, Givaldan A, Brehelin M, Laumond C (1997). Symbiosis and pathogenicity of nematode-bacterium complexes. Symbiosis..

[123] Meusch D, Gatsogiannis C, Efremov RG, Lang AE, Hofnagel O, Vetter IR (2014). Mechanism of Tc toxin action revealed in molecular detail. Nature..

[124] Blackburn M, Golubeva E, Bowen D, Ffrench-constant RH (1998). A novel insecticidal toxin from *Photorhabdus luminescens*, toxin complex a (Tca), and its histopathological effects on the midgut of *Manduca sexta*. Am Soc Microbiol..

[125] Sheets JJ, Hey TD, Fencil KJ, Burton SL, Ni W, Lang AE (2011). Insecticidal toxin complex proteins from *Xenorhabdus nematophilus*: structure and pore formation. J Biol Chem..

[126] McInerney BV, Gregson RP, Lacey MJ, Akhurst RJ, Lyons GR, Rhodes SH (1991). Biologically active metabolites from *Xenorhabdus* spp. part 1 dithiolopyrrolone derivatives with antibiotic activity. J Nat Prod..

[127] McInerney BV, Gregson RP, Lacey MJ, Akhurst RJ, Taylor WC (1991). Biologically active metabolites from *Xenorhabdus* spp. part 2. Benzopyran-1-one derivatives with gastroprotective activity. J Nat Prod..

[128] Li J, Hu K, Webster JM (1998). Antibiotics from *Xenorhabdus* spp. and *Photorhabdus* spp. (enterobacteriaceae): (Review). Chem Heterocycl Compd..

[129] Dunphy GB, Webster JM (1984). Interaction of *Xenorhabdus nematophilus* subsp. *nematophilus* with the haemolymph of *Galleria mellonella*. J Insect Physiol..

[130] Akhurst RJ (1982). Antibiotic activity of *Xenorhabdus* spp. bacteria symbiotically associated with insect pathogenic nematodes of the families heterorhabditidae and steinernematidae. J Gen Microbiol..

[131] Sergeant M, Baxter L, Jarrett P, Shaw E, Ousley M, Winstanley C (2006). Identification, typing, and insecticidal activity of *Xenorhabdus* isolates from entomopathogenic nematodes in United Kingdom soil and characterization of the xpt toxin loci. Appl Environ Microbiol..

[132] Rodou A, Ankrah DO, Stathopoulos C (2010). Toxins and secretion systems of *Photorhabdus luminescens*. Toxins..

[133] Aktories K, Schmidt G, Lang AE (2014). *Photorhabdus luminescens* Toxins TccC3 and TccC5: insecticidal ADP-ribosyltransferases that modify threonine and glutamine. Curr Top Microbiol Immunol..

[134] Jallouli W, Zouari N, Jaoua S (2010). Involvement of oxidative stress and growth at high cell density in the viable but nonculturable state of *Photorhabdus temperata* ssp. *temperata* strain K122. Process Biochem..

[135] Forst S, Nealson K (1996). Molecular biology of the symbiotic-pathogenic bacteria *Xenorhabdus* spp. and *Photorhabdus* ssp,. Microbiol Rev..

[136] Duchaud E, Rusniok C, Frangeul L, Buchrieser C, Givaudan A, Taourit S (2003). The genome sequence of the entomopathogenic bacterium *Photorhabdus luminescens*. Nat Biotechnol..

[137] Waterfield N, Kamita SG, Hammock BD, Ffrench-Constant R (2005). The *Photorhabdus* Pir toxins are similar to a developmentally regulated insect protein but show no juvenile hormone esterase activity. FEMS Microbiol Lett..

[138] Seo S, Lee S, Hong Y, Kim Y (2012). Phospholipase A2 inhibitors synthesized by two entomopathogenic bacteria, *Xenorhabdus nematophila* and *Photorhabdus temperata* subsp. *temperata*. Appl Environ Microbiol..

[139] Stanley D, Kim Y (2011). Prostaglandins and their receptors in insect biology. Front Endocrinol.

[140] Shrestha S, Park Y, Stanley D, Kim Y (2010). Genes encoding phospholipases A2 mediate insect nodulation reactions to bacterial challenge. J Insect Physiol..

[141] Kim E, Jeoung S, Park Y, Kim K, Kim Y (2015). A novel formulation of *Bacillus thuringiensis* for the control of brassica leaf beetle, *Phaedon brassicae* (Coleoptera: Chrysomelidae). J Econ Entomol..

[142] Burke JE, Dennis EA (2009). Phospholipase A 2 structure/function, mechanism, and signaling. J Lipid Res..

[143] Stanley D, Kim Y (2014). Eicosanoid signaling in insects: from discovery to plant protection. CRC Crit Rev Plant Sci..

[144] Shrestha S, Kim Y (2008). Eicosanoids mediate prophenoloxidase release from oenocytoids in the beet armyworm *Spodoptera exigua*. Insect Biochem Mol Biol..

[145] Cerenius L, Lee BL, Söderhäll K (2008). The proPO-system: pros and cons for its role in invertebrate immunity. Trends Immunol..

[146] Cerenius L, Söderhäll K (2004). The prophenoloxidase-activating system in invertebrates. Immunol Rev..

[147] Crawford JM, Portmann C, Zhang X, Roeffaers MBJ, Clardy J (2012). Small molecule perimeter defense in entomopathogenic bacteria. Proc Natl Acad Sci USA.

[148] Bonifassi E, Fischer-Le Saux M, Boemare N, Lanois A, Laumond C, Smart G (1999). Gnotobiological study of infective juveniles and symbionts of *Steinernema scapterisci*: a model to clarify the concept of the natural occurrence of monoxenic associations in entomopathogenic nematodes. J Invertebr Pathol..

[149] Sicard M, Ramone H, Le Brun N, Pagès S, Moulia C (2005). Specialization of the entomopathogenic nematode *Steinernema scapterisci* with its mutualistic *Xenorhabdus* symbiont. Naturwissenschaften..

[150] Ensign JC, Lan Q, Dyer D. Mosquitocidal *Xenorhabdus*, lipopeptide and methods. United States, Patent Application Publication. https://patents.google.com/patent/US20140274880A1/en.

[151] Ji D, Yi Y, Kang GH, Choi YH, Kim P, Baek NI (2004). Identification of an antibacterial compound, benzylideneacetone, from *Xenorhabdus nematophila* against major plant-pathogenic bacteria. FEMS Microbiol Lett..

[152] Gill SS, Cowles EA, Patricia V (1992). *Bacillus Thuringiensis* endotoxins. Annu Rev Entomol..

[153] Shrestha YK, Jang EK, Yu YS, Kwon M, Shin JH, Lee KY (2011). Oral toxicity of symbiotic bacteria *Photorhabdus* spp. against immature stages of insects. J Asia Pac Entomol..

[154] Koenraadt CJM, Takken W (2003). Cannibalism and predation among larvae of the *Anopheles gambiae* complex. Med Vet Entomol..

[155] Eom S, Park Y, Kim H, Kim Y (2014). Development of a high efficient “Dual Bt-Plus” insecticide using a primary form of an entomopathogenic bacterium, *Xenorhabdus nematophila*. J Microbiol Biotechnol..

[156] Niyonsaba F, Iwabuchi K, Someya A, Hirata M, Matsuda H, Ogawa H (2002). A cathelicidin family of human antibacterial peptide LL-37 induces mast cell chemotaxis. Immunology..

[157] Bravo A, Gill SS, Soberón M (2008). Mode of action of *Bacillus thuringiensis* Cry and Cyt toxins and their potential for insect control. Nucl Inst Methods Phys Res A..

[158] Boudko DY, Moroz LL, Linser PJ, Trimarchi JR, Smith PJS, Harvey WR (2001). *In situ* analysis of pH gradients in mosquito larvae using non-invasive, self-referencing, pH-sensitive microelectrodes. J Exp Biol..

[159] Chen J, Aimanova KG, Fernandez LE, Bravo A, Soberon M, Gill SS (2009). *Aedes aegypti* cadherin serves as a putative receptor of the Cry11Aa toxin from *Bacillus thuringiensis* subsp. *israelensis*. Biochem J..

[160] Chen J, Aimanova KG, Pan S, Gill SS (2009). Identification and characterization of *Aedes aegypti* aminopeptidase N as a putative receptor of *Bacillus thuringiensis* Cry11A toxin. Bone..

[161] Kajla MK, Barrett-Wilt GA, Paskewitz SM (2019). Bacteria: a novel source for potent mosquito feeding-deterrents. Sci Adv..

[162] Walther CJ, Couche GA, Pfannenstiel MA, Egan SE, Bivin LA, Nickerson KW (1986). Analysis of mosquito larvicidal potential exhibited by vegetative cells of *Bacillus thuringiensis* subsp. *israelensis*. Appl Environ Microbiol..

[163] Patil CD, Patil SV, Salunke BK, Salunkhe RB (2012). Insecticidal potency of bacterial species *Bacillus thuringiensis* SV2 and *Serratia nematodiphila* SV6 against larvae of mosquito species *Aedes aegypti*, *Anopheles stephensi*, and *Culex quinquefasciatus*. Parasitol Res..

